# Entomological Exploration of Sand Flies in Human Communities Affected by Cutaneous and Visceral Leishmaniasis in El Hajeb Province, Morocco

**DOI:** 10.1155/2023/4628625

**Published:** 2023-04-27

**Authors:** Karima El-Mouhdi, Mohammed Fekhaoui, Abdelkader Chahlaoui, Smahane Mehanned, Chafika Faraj, Samia Boussaa

**Affiliations:** ^1^Ministry of Health and Social Protection, Higher Institute of Nursing Professions and Health Techniques, Meknes, Morocco; ^2^Scientific Institute, Mohammed V University, Rabat, Morocco; ^3^Faculty of Sciences, Moulay Ismail University, Meknes, Morocco; ^4^National Institute of Hygiene, Medical Entomology, Ministry of Health and Social Protection, Rabat, Morocco; ^5^Ministry of Health and Social Protection, Higher Institute of Nursing Professions and Health Techniques, Fez, Morocco

## Abstract

Sand flies are the exclusive vectors of leishmaniasis. This group of parasitic diseases is a serious public health problem in Morocco. The aim of this study was to investigate the sand fly fauna, mainly the species composition, biodiversity, and seasonal activity of sand flies in El Hajeb in central Morocco. A total of six stations (Aït Naaman, Aït Rbaa, Aït Brahim, Ain Taoujdate, Sidi Mbarek, and Aït Oufella) were studied, five of which had recently recorded cases of leishmaniasis. Sand fly bimonthly captures were carried out using a sticky paper trap in different biotopes from March to December 2019. A total of 14590 adult sand flies were collected. The activity of the sand fly started in April and declined in November. The periods of high abundance were July, September, and November. Morphological identification of sand flies shows the presence of twelve species: *Phlebotomus papatasi*, *P. longicuspis*, *P. perniciosus*, *P. sergenti*, *P. bergeroti*, *P. alexandri*, *P. dreyfussi*, *P. ariasi*, *Sergentomyia fallax*, *S. minuta*, *S. schwetzi*, and *S. antennata*. The analysis showed that species belonging to the genus *Phlebotomus* were the most dominant (93.3%) and the risk periods were spread during the summer and autumn seasons. The present study provides for the first time information on the species of sand flies in El Hajeb. It, therefore, provides decision makers with an important tool to conduct vector control actions during peak periods in order to limit the transmission of leishmaniasis. A preprint was made available by the research square in the following link: “https://assets.researchsquare.com/files/rs-1409330/v1/dfef7013-0327-4a54-897f-214924a2d950.pdf?c=1646838874.”

## 1. Introduction

Sand flies (Diptera, Psychodidae, and Phlebotominae) are widely distributed in tropical and temperate climates and are of great medical and veterinary importance. They are currently considered as the exclusive vectors of human and canine leishmaniasis [1]. These diseases are ranked second only to malaria as vector-borne diseases threatening global health [2]. They represent a complex group of parasitic diseases, both clinically and ecoepidemiologically [1, 2].

In Morocco, leishmaniasis is a serious health problem and is endemic in several regions of the country, manifesting itself in two main clinical forms: cutaneous leishmaniasis (CL) and visceral leishmaniasis (VL) [3, 4].

From the epidemiological point of view, the main foci of the visceral and cutaneous forms, which are caused by the *Leishmania infantum* (*L. infantum*) parasite, have been recorded mainly in the north of the country, while the outbreaks of the wet cutaneous forms, which are caused by *Leishmania major* (*L. major*), were reported in the south of the country; and those of the dry cutaneous forms, which are caused by *Leishmania tropica* (*L. tropica*), were reported mainly in the center of the country [3, 5].

Beyond this clinical and parasitic variety of leishmaniasis, their common point is that they are transmitted in natural conditions by the bite of different species of infected female sand flies [1]. These insects are called “*Chniwla*” in most regions in Morocco [6–8].

From an entomological point of view, not only the vectorial capacity of sand flies of the genera *Phlebotomus* in the old world and *Lutzomyia* in the new world has been demonstrated for the transmission of leishmaniasis but also that of other viral or bacterial diseases [1].

In contrast, in Morocco, five species of the *Phlebotomus* genus are involved in the transmission of leishmaniasis. The first one belonging to the *Phlebotomus* (*P*) subgenus is *P. papatasi* incriminated in the transmission of *L. major*; the second species belonging to the subgenus Paraphlebotmus is *P. sergenti* which is responsible for the transmission of *L. tropica*; the last three species belonging to the subgenus Larroussius are *P. perniciosus*, *P. ariasi,* and *P. longicuspis*. These three species are involved in the transmission of *L. infantum*, the causative agent of sporadic cutaneous forms and the severe visceral forms.

From a strategic point of view, a National Program for the Control of Leishmaniasis (NPCL) has been designed since 1997 to control the epidemiological situation of leishmaniasis in the country. Currently, its main goal is to eliminate all forms of leishmaniasis by 2030, based on a set of strategic axes: (a) free therapeutic management of cases; (b) control of the animal reservoir, particularly dogs and rodents; (c) vector control; (d) continuous training of health personnel; and (e) intersectoral collaboration and information, education, and communication [3, 9].

Thus, the present study is part of the entomological surveillance and vector control of the NPCL. It aims to provide decision makers with basic data on the phlebotomine fauna circulating at the level of the province of El Hajeb in the center of Morocco where autochthonous cases of cutaneous and visceral leishmaniasis have been recorded.

## 2. Methods

### 2.1. Study Area

**Figure 1:**
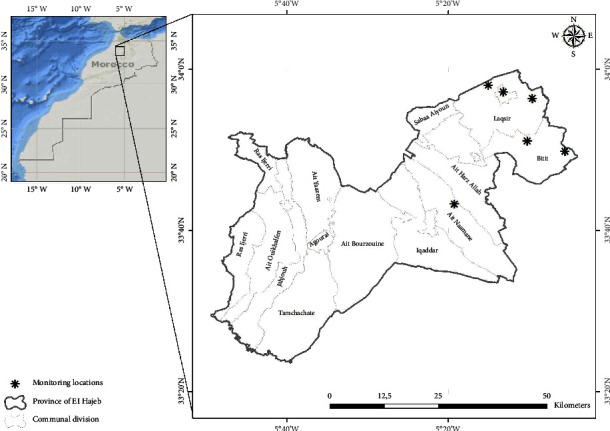
Map showing the sand fly collection stations in El Hajeb [10].

The entomological study was carried out in six localities of the province of El Hajeb: the stations of Aït Naaman (05°19′13.79″ W; 33°43′15.38″ N), Aït Rbaa (05°10′10.79″ W; 33°51′04.67″ N), and Aït Brahim (05°05′28.75″ W; 33°49′48.46″ N) located northwest of El Hajeb and the stations of Ain Taoujdate (05°13′07.78″ W; 33°57′09.33″ N), Sidi Mbarek (05°15′01.50″ W; 33°57′59.60″ N), and Aït Oufella (05°09′31.93″ W; 33°56′20.51″ N) located north of the province (Figure 1).

The climate of El Hajeb is semihumid of the Mediterranean type; it is characterized by a hot and dry summer and a mild and rainy winter. The average annual rainfall is about 572 mm and the average annual temperature is about 17°C with a maximum of 35°C in July and a minimum of 4°C in January [11].

The choice of the trapping stations was based on epidemiological justifications. Indeed, we based our work on retrospective studies of the epidemiological situation of leishmaniasis in this province during the last five years [12]. These data allowed us to direct our survey efforts towards localities that have been affected by these vector-borne diseases.

The support of local authorities and the collaboration of community agents and inhabitants allowed us to agree to trap in six stations (five houses and one uncontrolled public landfill) in families with a history of leishmaniasis. These are the Sidi Mbarek station and the Aït Oufella station in the rural commune of Laqsir where autochthonous cases of cutaneous leishmaniasis (CL) and visceral leishmaniasis (VL) have been reported to the health authorities; the Aït Rbaa station in the rural commune of Bitit where cases of VL and CL have also been reported; the station of Aït Brahim where the surveys were carried out in the public landfill located near the locality where infantile VL cases were notified; the station of Aïn Taoujdate where cases of CL were declared; and the station of Aït Naaman in the commune of El Hajeb where no case of leishmaniasis was reported was chosen as a control station.

### 2.2. Trapping Technique and Specimen Collection

The sampling of sand flies took place during the year 2019 in six stations in the province of El Hajeb in central Morocco. For the collection of sand flies, we used sticky paper traps (white A4 papers, soaked in castor oil on both sides) during the whole night [13]. During each capture session, 40 sheets were used and deposited in the afternoon at sunset and recovered the next morning at sunrise. These sticky traps were placed in the same location throughout the trapping period. Sand flies were captured in each locality on a bimonthly basis throughout the survey period. The surveys were planned for one year from April 2019 to January 2020, April to January being the period of activity of sand flies in Morocco [14].

The main biotopes that were surveyed were manure and animal burrows, chicken houses, stables, and near vegetation and rocks, as well as holes and inside and outside the house. Thus, we also equipped a mini mobile microscope adapted to our smartphone to photograph the sand flies captured in the field.

### 2.3. Laboratory Work

The collected sand flies were transported to the laboratory where they were sorted, counted, sexed, and preserved in 70% ethanol solution. The sand flies were thinned with a Marc-André solution and mounted between the slide and coverslip and then placed under the microscope for morphological identification using the sand fly identification keys published by the Ministry of Health [3], based on the pharynx and external genitalia in males and the spermatheca and cibarium in females. Thus, the identification of sand flies of the *Larroussius* subgenus was also carried out by referring to Leger and Depaquit [15] and Boussaa [16] and those of the subgenus Paraphlebotomus subgenus following Depaquit's method [17, 18].

### 2.4. Data Analysis

Based on the available sources, references, and records, the sand fly fauna has never been studied before in El Hajeb. The characteristics of the captured fauna were determined by calculating six parameters, namely,

(i) Abundance (A) which represents the total collected count of each species.
(ii) The relative abundance of each species (RA) is estimated by dividing the number collected of a given species by the total count of all species collected multiplied by 100:
  (1) $$\begin{array}{c} RA=\frac{n}{N\times 100}\text{.} \end{array}$$
(iii) Species richness (S) represents the total number of species in the stand.
(iv) The diversity of the phlebotomine stand is expressed by the Shannon diversity index “*H*′” and the Evenns equitability index “*E*,” where
  (2) $$\begin{array}{c} H^{\prime }=-⅀PiLnPi\text{,} \end{array}$$
where Pi is the proportion of the total number of species samples
  (3) $$\begin{array}{c} E=\frac{H^{\prime }}{H\max}\text{,} \end{array}$$
where *H*max:LnS.
(v) Sex ratio (SR) is the ratio of males to females:
  (4) $$\begin{array}{c} SR=\frac{M}{F}\text{.} \end{array}$$
(vi) The density (D) is represented by the number of individuals per unit area of oil paper. It is expressed as the number of sand flies per square meter per night:
  (5) $$\begin{array}{c} D=\frac{ph}{s/night}\text{,} \end{array}$$
where *D*: density; Ph: number of sand flies; and *S*: surface.

## 3. Results and Discussion

From an ecoepidemiological point of view, the province of El Hajeb constitutes a very interesting field of study because of its geographical proximity to epidemic foci of cutaneous leishmaniasis in the provinces of Sefrou [19] and Moulay Yacoub [20]. In addition, recent studies have also shown the existence of autochthonous cases of CL and VL in the province of El Hajeb [12] whose responsible vectors are not yet recognized. Indeed, entomological data do not exist on the phlebotomine fauna circulating in this region. However, the identification of the species responsible for the transmission of leishmaniasis diseases in the affected localities is a fundamental step for the vector control activities and the prevention of these diseases, specifically the severe forms that threaten humans and dogs [21]. It is in this perspective that the present work was carried out, exploring for the first time the existing fauna and identifying the species incriminated in the transmission of this disease.

### 3.1. Diversity, Species Richness, and Sex Ratio of Sand Flies Caught

A total of 14950 sand flies were captured, of which 78.3% (11715) were males and 21.7% (3235) were females. The abundance, diversity, species richness, and sex ratio of the sand fly population captured at the six stations are presented in Table 1.

Across the region, twelve species were identified with a difference between stations, and the sex ratio was 4.07 : 0.25. In fact, the ratio of males to females was in favor of males in the stations of Aït Taoujdate (2.8 : 0.36), Sidi Mbarek (4.04 : 0.25), Aït Oufella (8.23 : 0.12), Aït Rbaa (2.85 : 0.35), and Aït Naaman (1.95 : 0.52). In contrast, in the locality of Aït Brahim, females were collected more than males (0.73 : 1.35). In addition, the diversity of sand flies in El Hajeb was calculated using the Shannon diversity index “*H*′” and Evenns equitability index “*E*” (Table 1). The fauna of the localities of Aït Rbaa and Sidi Mbarek had the greatest biodiversity (11 and 12 species out of 12), while that of Aït Brahim showed the least biodiversity (7 species out of 12).

The analysis of the Shannon diversity index shows that the *H*′ values in the stations of Aït Naaman (*H*′ = 1.75) and Aït Brahim (*H*′ = 1.54) are closer to each other, while they are different in terms of maximum value of diversity. This means that the distribution of species at the level of the station of Aït Brahim in spite of containing fewer species (7 species) is better than that in the station of Aït Naaman (10 species).

Thus, the values of the diversity index in all the stations studied are a little different from each other with a diversified population over the whole study area. This indicates the presence of a more stable ecosystem at El Hajeb. It should also be noted that the most diversified station was that of Aït Naaman with a value of *E* = 0.76. This is due to an approximation between its diversity value *H* and its maximum theoretical value *H*′. This explains why the reproductive potential of the phlebotomine population seems to be the same, while the lowest diversity value was recorded in the station of Aït Oufella with a value of *E* = 0.265.

### 3.2. Inventory and Relative Abundance of the Identified Sand Flies Species

Of the 14590 sand flies collected, 14070 were identified morphologically, and the rest of the sand flies (520) gravid females were retained for possible future molecular identification. The detailed results of the identification of 14070 sand flies are presented in Table 2.

In fact, among 14070 identified sand flies, 93.3% (13128) specimens belonged to the genus *Phlebotomus* and 6.7% (942) to the genus *Sergentomyia*. In addition, the morphological identification of the collected specimens revealed the presence of 12 species: seven species of the genus *Phlebotomus* and four species of the genus *Sergentomyia*: *P. papatasi*, *P. bergeroti*, *P. sergenti*, *P. alexandri*, *P. longicuspis*, *P. perniciosus*, *P. ariasi*, *S. minuta*, *S. schwetzi*, *S. fallax*, *S. antennata*, and *S. dreyfussi*.

On the other hand, 24 species of sand flies have been described in Morocco, five of which are known for their vectorial capacities to transmit the leishmaniasis which represents a public health problem [6, 22]. These species are (a) *P. papatasi*, the vector of CL *major* which is widespread in the south and southeast of the country [3, 23, 24], (b) *P. sergenti* which is responsible for the transmission of cutaneous leishmaniasis to *L. tropica* for which most of the recorded cases are reported in the center of the kingdom [24, 25], and (c) the other three species are *P. perniciosus*, *P. ariasi,* and *P. longicuspis* which have been proven as vectors of severe forms of the disease, especially VL for which most cases have been reported in the north [3, 26].

In our study, 12 species were identified, which represents 50% of the Moroccan sand fly species. Among these 12 species, five were considered as vectors of the disease. They were also collected in the region of El Hajeb and represent 91.6% of the sand flies captured and are present in 83% of the sites surveyed (5/6). These include *P. sergenti*, *P. papatasi*, *P. longicuspis*, *P. ariasi*, and *P. perniciosus*.

On the other hand, the analysis of the results obtained showed that there is cohabitation between the 3 species: *P. papatasi*, *P. sergenti*, and *P. longicuspis*. Although the province of El Hajeb is located in the center of Morocco, this coexistence was also noted by Boussaa et al. [26] in their study conducted in the south and north of the country.

In another aspect, the analysis of the results in terms of abundance for each station revealed that the most important abundance was recorded at the station of Aït Oufella with 6833 specimens (46.8%), the station of Aïn Taoujdate with 3023 specimens (20.7%), and the station of Aït Rbaa with 2083 specimens (14.3%), then the station of Aït Naaman with 1044 (9.6%) and of Sidi M'barek with 1407 specimens (7.2%), and finally the station of Aït Brahim with 200 specimens (1.4%). However, it was noticed that the abundance of fauna was very important compared to the low incidence of leishmaniasis recorded in the region [12]. This finding has been found in other studies in other regions of Morocco [27–30]. This is mainly related to the ecology of the parasite rather than to the distribution of the vector [30]. Thus, recent studies have shown that the sand fly is called “*Chniwla*” by the local population and by health professionals in the region [7–9].

### 3.3. Seasonal Activity and Monthly Fluctuation of Fauna in El Hajeb

**Figure 2:**
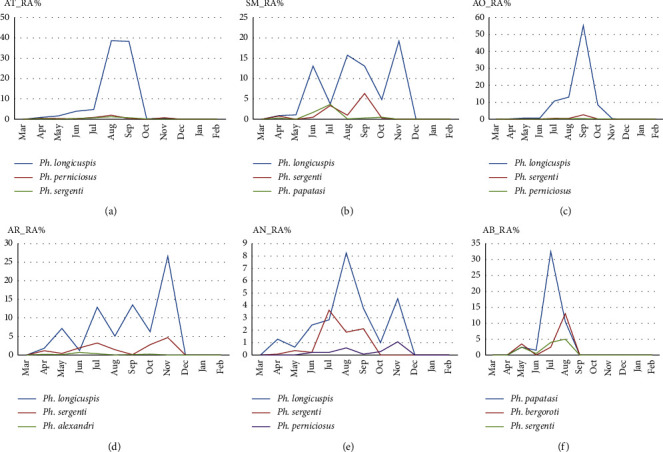
Monthly activity of the sand fly fauna in the six stations of El Hajeb in 2019.

The results of the monthly activity of the three most abundant species in the six stations, Ain Taoujdate (AT), Sidi Mbarek (SM), Aït Oufella (AO), Aït Rbaa (AR), Aït Naaman (AN), and Aït Brahim (AB), are presented in Figure 2. Indeed, differences have been noticed in the monthly evolution of each species.

At the Aïn Taoujdate station, species of the genus *Phlebotomus*, particularly of the subgenus *Larroussius*, were the most abundant. Although the peak of activity occurred in August and September, seasonal activity lasted for two seasons, starting in April in the early spring and ending in October in the late summer, indicating that the life span of sand flies is relatively long, allowing sand flies to reproduce once or twice during this period. Indeed, the results show that the seasonal abundance of adults of *P. longicuspis* recorded a significant peak in activity during the summer months of July, August, and September. Similarly, the species *P. perniciosus* and *P. sergenti* showed a similar pattern, but with a single peak in August (Figure 2(a)).

For the Sidi Mbarek station, *P. longicuspis*, *P. sergenti*, and *P. papatasi* were the most commonly encountered species. The seasonal activity of adults of *P. longicuspis* reflected three peaks: a first peak in June and then the number of sand flies decreased steadily in July as the climate became warmer and then increased slightly to mark a second peak at the end of September and October, but the last peak was more important than the previous one in November, while the monthly abundance of *P. sergenti* and *P. papatasi* showed a bimodal peak pattern: one in July and the other in September which corresponds to the hot summer season that is favorable for sand fly reproduction (Figure 2(b)).

Concerning the station of Aït Oufella, the analysis of the monthly seasonal activity of the identified sand flies showed that the most encountered species belonged to the genus *Phlebotomus*, in particular *P. longicuspis,* which showed a bimodal variation, the first peak being in July and the second in September, whereas *P. sergenti* and *P. perniciosus* showed only one peak in September when the climate is warm (Figure 2(c)). It seems therefore that at this station, the summer season is the best breeding period for the different species of sand flies.

At the Aït Rbaa station, the sand fly activity ranged from April to November. The seasonal abundance of adults of *P. longicuspis* peaked in May, and then, the number of sand flies increased slightly in July and September as the climate warmed up, reaching its peak in November. Adult *P. sergenti* activity followed a similar pattern, but peaked at the end of April, then in July, and finally in November (Figure 2(d)). This means that the summer period offers more opportunities for sand flies to breed since activity covers the months of July, August, and September, indicating that the life span of the sand fly is longer in this season compared to autumn when the sand fly activity is limited only to the month of November.

At the Aït Brahim station, the sand fly activity started in May and the adults of *P. papatasi* showed a single peak in July, while adults of *P. bergeroti* also showed a single peak but in August (Figure 2(f)).

In the majority of the locations prospected, sand flies started to appear in April and disappeared by the end of November. This could be explained by the moderate climate of the El Hajeb region which is of the Mediterranean type characterized by a hot summer offering sand flies the possibility of breeding during the whole period of July-August-September, whereas when the climate became colder in autumn, the life span of the sand flies is short with a single peak of activity in November.

**Figure 3:**
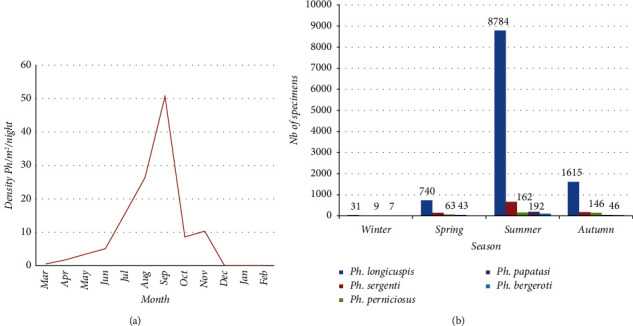
Total evolution of the seasonal activity of the sand fly fauna in El Hajeb in 2019.

**Figure 4:**
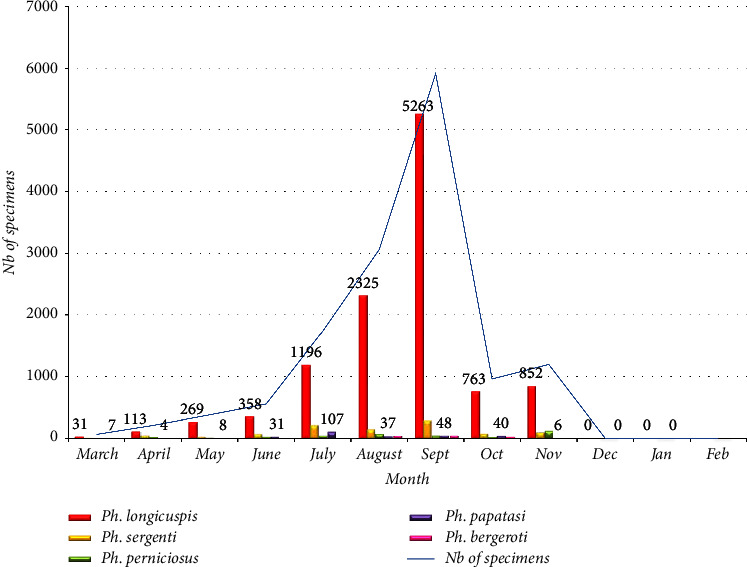
Total monthly fluctuation of sand flies in El Hajeb province in 2019.

On the whole, the analysis of the monthly and seasonal activities of the sand flies population of the entire region of El Hajeb shows that the maximum total density was recorded in the summer and autumn seasons, particularly in the months of July, August, September, and November, density which stood at, respectively, 31 *ph*/m^2^/night, 53 *ph*/m^2^/night, 101 *ph*/m^2^/night, and 21 *ph*/m^2^/night (Figures 3 and 4).

The monthly activity of the five most abundant species incriminated in the transmission of leishmaniasis in El Hajeb province (*P. longicuspis* (79.4%), *P. sergenti* (7.2%), *P. perniciosus* (2.7%), *P. papatasi* (2.0%), and *P. bergeroti* (1.1%)) ranged from March to November. The highest abundance was recorded during the months of July, August, September, and November. The total catches of sand flies during the year show that there are two peaks: the first and most important one was in summer when the climate is warm and the second one in autumn.

**Figure 5:**
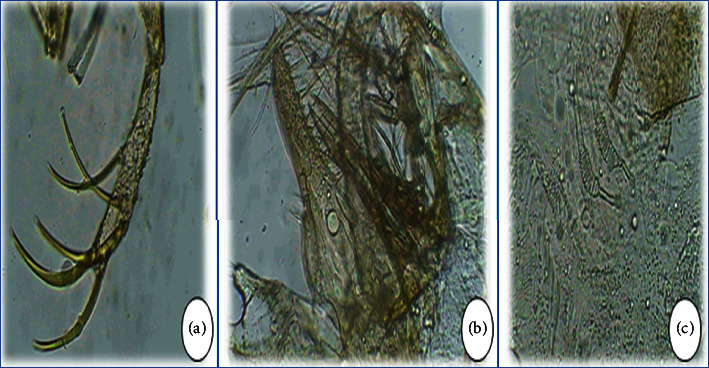
*Phlebotomus longicuspis*: (a, b) style and pointed copulatory valves of males; (c) spermathecae of female.

An analysis of monthly seasonal species-specific activity (Figure 4) shows that *P. longicuspis* (Figure 5) was the most abundant in all stations, except the station of Ait Brahim. This species represents 79.7% of the sand fly population in the region of El Hajeb, and its activity extends from April to November with three peaks. The first peak begins in June, and the number of sand flies continues to increase to reach its maximum in September where it will mark its second peak which is the most important, and then, the number decreases steadily in October and increases slightly to record a third peak that was less important in November. Indeed, this species was found in all stations and during the whole study period but with very important abundance in the two localities of Aïn Taoujdate and Aït Oufella.

On the other hand, the long period of activity and high abundance of these confirmed VL vector species represent a threat and indicate the potential risk of VL transmission in the said province. These results corroborate the study of Al-Koleeby et al. [31] in which the most important peak is located in September, but diverge on the other hand in which this species can show a pattern with a single peak as is the case of the province of Chichaoua [32] or with a pattern of two peaks as is the case of the province of Zagora [31].

Similarly, this species showed a three-peak pattern in our study site. This can be explained by the ability of *P. longicuspis* to adapt to the environmental conditions of each region. In addition, 53% of these species were captured in the locality of Aït Oufella whose altitude is 581 m. In this context, Guernaoui et al. [29] showed that this species is very abundant between the altitudes of 600 m and 799 m.

**Figure 6:**
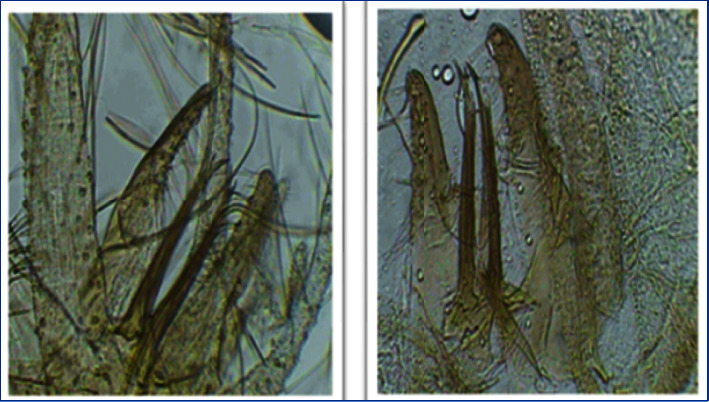
*Phlebotomus perniciosus* of typical shape with a bifid apex of the male penis.

Concerning *P. perniciosus*, it should first be noted that the morphological identification of this species showed that almost all captured males are in typical form with a bifid apex penis (Figure 6). This species was captured from April to November, and its seasonal trend reflects a bimodal pattern with two peaks (Figure 4). The first peak was in July-August and the second peak was in November. In addition, this species was not found in the Aït Brahim station although it was collected during the entire study period in the other stations. In this context, studies conducted in the vicinity of El Hajeb have revealed that the seasonal evolution of this species takes the form of two peaks [19].

**Figure 7:**
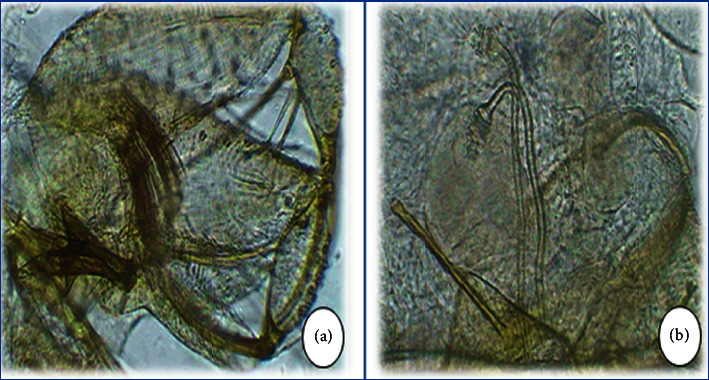
*Phlebotomus sergenti*: (a) male paramere; (b) female spermathecae.

The species *P. sergenti* (Figure 7) was encountered during the entire study period since this species is the only proven vector of *L. tropica* in Morocco. It was also caught in all stations and with very high abundance in the localities of Aït Oufella and Aït Rbaa. Its period of activity ranges from April to November with a biphasic pattern (Figure 4), the first one in June-July and the other one in September. Our results confirm those found in the province of Fez [33] which revealed that the seasonal activity of this species is bimodal.

**Figure 8:**
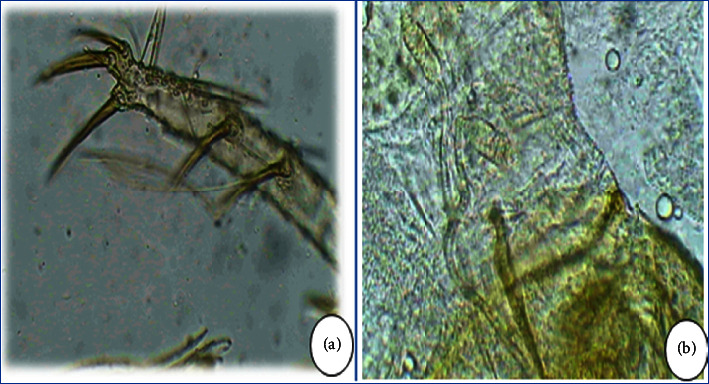
*Phlebotomus papatasi*: (a) slender style with five terminal spines of male; (b) spermathecae of female.

The species *P. Papatasi* (Figure 8) showed a monophasic pattern with a single peak in July (Figure 2). This vector species of *L. major,* especially in southern Morocco and which has long been considered adapted to the arid climate [24], is also adapted to the temperate climate that prevails in the center of the country since our results show the presence of this species from April to November and in the majority of stations (5/6), but with a high abundance in the stations of Aït Oufella and Aït Brahim. This result was also revealed by studies conducted in the provinces near El Hajeb [19] where it was also collected from April to November.

**Figure 9:**
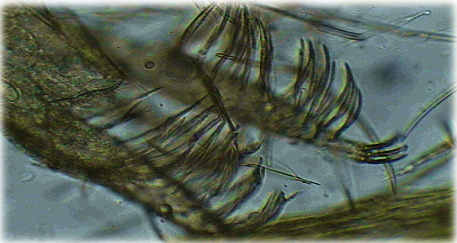
*Phlebotomus bergeroti* with a malformation at the level of the terminal spines.

On the other hand, other species belonging to the genus *Phlebotomus* were encountered in El Hajeb but with weak abundance. This is the case in particular for *P. ariasi*, *P. alexandri,* and *P. bergeroti*. The latter was caught with malformations for which the males were found to have a three-lobed paramere with a larger upper lobe and a lateral lobe with 3 spatulate terminal spines instead of 2 spines (Figure 9).

**Figure 10:**
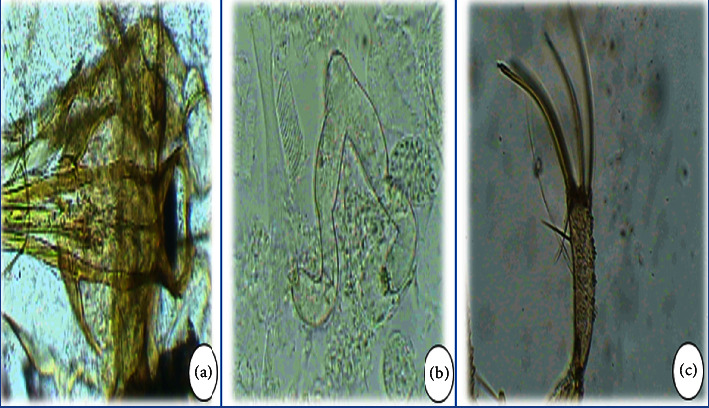
*Sergentomyia minuta*: (a) cibarium of female; (b) spermathecae of female; and (c) style with nondeciduous silk of male.

From another perspective, sand flies of the genus *Sergentomyia* represent 6.7% of the specimens collected, belonging to two subgenera: *Sergentomyia* and *Grassomyia*. These are *S. minuta* (Figure 10), *S. schwetzi*, *S. fallax*, *S. antennata*, and *S. dreyfusi*. These species prefer, according to Boussaa et al. [34], the altitudes between 800 m and 1000 m. Similarly, Guernaoui et al. [29] state that only the species *S. minuta* persists at the altitudes of 1200–2000 m. This was confirmed in our study particularly in the station of Aït Naaman which is located at an altitude of 1150 m and whose inventory of sand flies captured in this station revealed that the most abundant species belonged to the genus *Sergentomyia,* notably *S. minuta* (Figure 10), which represents 65.9%.

The activity period of *S. minuta* ranges from April to November with a bimodal pattern in two peaks, the first one which is the most important in August and the second one in September (Figure 10). This species was most abundant in the locality of Aït Naaman where we collected 90.2% of the total number of these species.

Within this framework, it is very useful to point out that the selection of the station of Aït Naaman was carried out to compare the fauna of this station where no case of leishmaniasis was declared with the other stations affected by the said disease. In this framework, our results show that most of the specimens met in this station are of the *Sergentomyia* genus which explains the absence of leishmaniasis cases in this locality. Indeed, the species of the *Sergentomyia* genus are not yet proven to be involved in the transmission of leishmaniasis [1, 4, 35]. Nevertheless, the absence of leishmaniasis cases should not eliminate the potential risk of transmission since the vector species of *L. infantum* (*P. longicuspis*) and *L. tropica* (*P. sergenti*) have also been found. It appears that the inventory of phlebotomine fauna in El Hajeb was very important in terms of quantity and quality. The majority of the species collected are proven to be involved in the transmission of the leishmaniasis disease, notably species belonging to the subgenus: (a) *Larroussius*, especially the species *P. longicuspis*, *P. perniciosus*, and *P. ariasi* which are responsible for the transmission of *Leishmania infantum,* the agent responsible for the severe visceral form, and the sporadic cutaneous form; (b) the subgenus *Paraphlebotomus* mainly *P. sergenti* vector incriminated in the transmission of anthropogenic CL *tropica*; and (c) the species of the subgenus *Phlebotomus* especially P. papatasi which is proven to be a vector of zoonotic CL major.

## 4. Conclusions

In summary, this study presents for the first time the circulating sand fly fauna in the El Hajeb region of central Morocco. It provides basic data on the abundance, biodiversity, seasonality of sand flies, and their risk periods of disease transmission. It was shown that the species of sand flies involved in the transmission of leishmaniasis (*P. papatasi*, *P. sergenti*, *P. longicuspis*, and *P. perniciosus*) occupy the first place in terms of predominance. These results serve as a very important tool for the decision makers of the National Leishmaniasis Control Program to focus their vector control actions towards the periods of high risk of transmission which coincide with the periods of peak density, especially in the summer and autumn seasons. Thus, entomological surveillance must be accompanied by awareness-raising and information activities for the inhabitants to avoid the health threats presented by sand flies and prevent the spread of leishmaniasis to other localities of El Hajeb in central Morocco.

## Figures and Tables

**Table 1:** Specific richness, diversity, abundance, and sex ratio of the fauna captured in the six stations studied in the El Hajeb region in 2019.

Station/ecocharacteristics	Ain Taoujdate	Sidi Mbarek	Aït Oufella	Aït Rbaa	Aït Naaman	Aït Brahim
Environment	Periurban	Rural	Rural	Rural	Periurban	Rural

Altitude (m)	470	431	581	922	1150	704

Abundance	3023	1044	6833	2083	1407	200

Male	2227	837	6093	1542	931	85

Female	796	207	740	541	476	115

Sex ratio	2.8 : 0.36	4.04 : 0.25	8.23 : 0.12	2.85 : 0.35	1.95 : 0.52	0.73 : 1.35

Species richness	9	12	10	11	10	7

*H*′	0.596	1.173	0.611	0.894	1.751	1.539

*H*max	2.197	2.485	2.303	2.398	2.303	1.946

*E*	0.271	0.472	0.265	0.373	0.76	0.791

Biotopes	(1) House and henhouse	(1) Domestic	(1) Manure	(1) Domestic	(1) Hen house	(1) Solid waste
	(2) Cow and sheep pen	(2) Stables	(2) Pens for cows, horses, and sheep	(2) Sheep pen	(2) Pen for horses and sheep	
	(3) Peridomestic	(3) Peridomestic	(3) Peridomestic rock	(3) Peridomestic	(3) Domestic perimeter	
		(4) Rocks				

Dominant species	(1) *P. longicuspis*	(1) *P. longicuspis*	(1) *P. longicuspis*	(1) *P. longicuspis*	(1) *S. minuta*	(1) *P. papatasi*
	(2) *P. perniciosus*	(2) *P. sergenti*	(2) *P. sergenti*	(2) *P. perniciosus*	(2) *P. longicuspis*	(2) *P. sergenti*
	(3) *P. sergenti*	(3) *P. papatasi*	(3) *P. perniciosus*	(3) *P. sergenti*	(3) *P. sergenti*	(3) *P. bergeroti*
		(4) *S. minuta*	(4) *P. papatasi*			

*H*′: Shannon–Wiener diversity index; *E*: Evenns equitability index.

**Table 2:** Inventory and relative abundance of phlebotomine species identified in the six stations studied in the El Hajeb region (2019).

Genera		*Phlebotomus*							*Sergentomyia*					Total
Subgenus		*Phlebotomus*		*Paraphlebotmus*		*Larroussius*			*Sergentomyia*				*Grassomyia*	
Species/station		*P. papatasi*	*P. bergeroti*	*P. sergenti*	*P. alexandri*	*P. longicuspis*	*P. perniciosus*	*P. ariasi*	*S. minuta*	*S. schwetzi*	*S. fallax*	*S. antennata*	*S. dreyfusi*	
Ain Taoujdate	M	21	14	68	12	2000	105	0	3	3	1	0	0	2227
	F	1	3	35	5	631	44	0	6	1	1	0	0	727
	Nb	22	17	103	17	2631	149	0	9	4	2	0	0	2954
	RA%	0.74	0.58	3.49	0.58	89.1	5.04	0	0.3	0.14	0.07	0	0	100

Sidi Mbarek	M	35	16	85	5	651	9	2	18	1	4	5	6	837
	F	21	5	49	3	73	10	0	9	0	0	0	1	171
	Nb	56	21	134	8	724	19	2	27	1	4	5	7	1008
	RA%	5.56	2.08	13.3	0.79	71.8	1.88	0.2	2.68	0.1	0.4	0.5	0.69	100

Aït Oufella	M	62	63	222	22	5582	107	8	5	5	17	0	0	6093
	F	46	2	82	5	373	18	1	4	2	6	0	0	539
	T	108	65	304	27	5955	125	9	9	7	23	0	0	6632
	RA%	1.63	0.98	4.58	0.41	89.8	1.88	0.14	0.14	0.11	0.35	0	0	100

Aït Rbaa	M	3	8	284	29	1187	11	0	3	3	7	5	2	1542
	F	6	0	44	6	365	6	0	7	0	0	1	1	436
	Nb	9	8	328	35	1552	17	0	10	3	7	6	3	1978
	RA%	0.46	0.4	16.6	1.77	78.5	0.86	0	0.51	0.15	0.35	0.3	0.15	100

Aït Naaman	M	0	0	97	7	267	31	15	335	82	44	5	48	931
	F	0	0	19	1	81	3	2	225	40	10	1	10	392
	Nb	0	0	116	8	348	34	17	560	122	54	6	58	1323
	RA%	0	0	8.77	0.6	26.3	2.57	1.28	42.3	9.22	4.08	0.45	4.38	100

Aït Brahim	M	33	26	13	0	0	0	0	4	4	5	0	0	85
	F	61	12	11	0	4	0	0	2	0	0	0	0	90
	Nb	94	38	24	0	4	0	0	6	4	5	0	0	175
	RA%	54	22	14	0	2.3	0	0	3.4	2.3	2.9	0	0	100

Total		289	149	1009	95	11214	344	28	621	141	95	17	68	14070

M: male; F: female; Nb: number of sand flies; RA: relative abundance.

## Data Availability

The data used to support the findings of this study are included within the article.
